# Cell fate clusters in ICM organoids arise from cell fate heredity and division: a modelling approach

**DOI:** 10.1038/s41598-020-80141-3

**Published:** 2020-12-29

**Authors:** Tim Liebisch, Armin Drusko, Biena Mathew, Ernst H. K. Stelzer, Sabine C. Fischer, Franziska Matthäus

**Affiliations:** 1 Faculty of Biological Sciences and Frankfurt Institute for Advanced Studies (FIAS), Goethe Universität Frankfurt am Main, Ruth-Moufang-Straße 1, 60438 Frankfurt, Germany; 2 Faculty of Biological Sciences and Buchmann Institute for Molecular Life Sciences (BMLS), Goethe Universität Frankfurt am Main, Max-von-Laue Str. 15, 60438 Frankfurt, Germany; 3 Center for Computational and Theoretical Biology (CCTB), Julius-Maximilians-Universität Würzburg, Campus Hubland Nord 32, 97074 Würzburg, Germany

**Keywords:** Developmental biology, Cell proliferation, Differentiation, Computational biology and bioinformatics, Computational models

## Abstract

During the mammalian preimplantation phase, cells undergo two subsequent cell fate decisions. During the first decision, the trophectoderm and the inner cell mass are formed. Subsequently, the inner cell mass segregates into the epiblast and the primitive endoderm. Inner cell mass organoids represent an experimental model system, mimicking the second cell fate decision. It has been shown that cells of the same fate tend to cluster stronger than expected for random cell fate decisions. Three major processes are hypothesised to contribute to the cell fate arrangements: (1) chemical signalling; (2) cell sorting; and (3) cell proliferation. In order to quantify the influence of cell proliferation on the observed cell lineage type clustering, we developed an agent-based model accounting for mechanical cell–cell interaction, i.e. adhesion and repulsion, cell division, stochastic cell fate decision and cell fate heredity. The model supports the hypothesis that initial cell fate acquisition is a stochastically driven process, taking place in the early development of inner cell mass organoids. Further, we show that the observed neighbourhood structures can emerge solely due to cell fate heredity during cell division.

## Introduction

The first steps during mammalian embryo development are ovulation and fertilisation, followed by the preimplantation phase. At this point, the blastocyst is formed, which later implants into the uterus^1^. Postimplantation development rapidly proceeds and involves multiple cell differentiation and morphological changes^1,2^. The first steps within the complex development processes in mammalian systems involve the cell fate decisions during the preimplantation phase. During development, the preimplantation phase is key to the success of pregnancy in mammals^3^. Despite this, processes taking place during this phase are not fully understood.

The mouse is a common model organism to study the preimplantation phase. The 8–16 cells morula is formed until embryonic day 2.5 (E2.5) after fertilisation. At this stage, the first cell fate decision is taking place. Cells on the surface of the morula become trophectoderm (TE), while cells inside the morula are forming the inner cell mass (ICM)^4,5^. During E3.0–E4.5, the second cell fate decision process takes place: ICM segregates into epiblast (Epi) and primitive endoderm (PrE)^6–8^. NANOG and GATA6 are described as the first markers for Epi and PrE segregation, respectively. Expression levels of NANOG and GATA6 undergo progressive changes during the morula stage and the early blastocyst^9,10^. In early blastocysts (E3.0), all ICM cells co-express NANOG and GATA6^7,11,12^. Subsequently, NANOG and GATA6 are gradually up- or down-regulated during the 32-cell stage. Thereby, both transcription factors repress each other locally^10,13–17^, leading to a mutually exclusive transcription factor expression in late blastocysts (64 cells)^18^. Once a cell-fate is determined it is only possible to switch the fate by an external modulation of the included signalling pathways^16,17,19^. While in the mid-point of blastocyst maturation (E3.5–E4.0), the spatial distribution of NANOG and GATA6 positive cells is commonly described as a salt-and-pepper pattern, GATA6 positive cells are sorted to the rim of the ICM in the late blastocyst (E4.5) and NANOG positive cells are forming an inner core^1,7,10,11,14^.

Three major processes contribute to the final cell fate arrangements in mid and late blastocysts (reviewed in Refs.^20,21^). (1) Inter- and intracellular chemical signalling involving the influence of NANOG, GATA6, and the FGF/ERK pathway onto the cell fate acquisition of single cells^10,13–15,17,19,22–29^. (2) Cell sorting processes: since cell lineage tracking revealed that ICM cells do not change their cell fate at E3.5, a cell sorting process is leading to the observed cell fate arrangements in the late blastocyst^7,10,30–33^. (3) Cell proliferation is hypothesised to contribute to the segregated state at E4.5^34–36^: from early blastocyst (E3.0) to late blastocyst (E4.5) it takes two to three rounds of cell divisions.

A recent study introduced a novel 3D stem cell system named *ICM organoids* (in the following the term *organoid* refers to the biological system, while the term *spheroid* is used in context of modelled data), which is based on inducible mouse embryonic stem cells^37^. ICM organoids mimic the segregation into Epi and PrE without forming a TE and reproduce key events and timing of cell fate specification and cell-cycle progression in the ICM of mouse blastocysts. Thus, ICM organoids provide a powerful tool to develop and test biological preimplantation hypotheses in vitro. The cell fate of the cells within the ICM organoids are determined by their expression level of the transcription factors NANOG and GATA6. In total, four different cell types are described: mostly NANOG expressing cells which express a small amount of GATA6 (N_+_G_−_), mostly GATA6 expressing cells which express a small amount of NANOG (N_−_G_+_), cells that express NANOG and GATA6 on a high level (N_+_G_+_) and cells that express both transcription factors at a low level (N_−_G_−_). After 24 hours (h) of growth, most ICM organoid cells are either N_+_G_−_ or N_−_G_+_, meaning most ICM organoid cells are expressing one of both transcription factors at a high level and the other one at a low level, respectively. The spatial segregation into an inner core of N_+_G_−_ cells and an outer layer of N_−_G_+_ cells is visible after 48 h of growth. However, in contrast to mid mouse blastocysts, which consist of approximately 64 cells, ICM organoids comprise over 400 cells after 24 h of growth and more than 1000 cells after 48 h of growth. It has been shown that the differentiation into N_+_G_−_ and N_−_G_+_ is mediated by cell-cell communication and the growth factor FGF4, resulting in robust and reproducible proportions in the ICM^38,39^. This process is robust to cell pertubations of the ICM, giving indication that ICM organoids are representing the mid mouse to late mouse blastocyst dynamics, despite their increased cell count. In order to quantify the patterns of neighbourhood distributions, a neighbourhood analysis of N_+_G_−_ cells, N_−_G_+_ cells, N_+_G_+_ cells and N_−_G_−_ cells was conducted^37^. The analysis revealed a local clustering of cells sharing the same expression type. A local clustering for N_+_G_+_ and N_+_G_−_ cells has also recently been shown for in vivo mouse embryos^40^.

The work of Mathew et al.^37^ indicates that between the two stages of an initial cell fate acquisition (E3.0–E3.5 early-blastocyst or 0 h old ICM organoids) and the final segregation of different cell fates (E4.5 late blastocyst or 48 h old ICM organoids), a local clustering of cell fates arises (E3.75 mid blastocyst or 24 h old ICM organoids). In order to test whether this pattern can be achieved through simple rules, static models were used. In these models, the cells were assigned to a cell fate but did not move. Four different hypotheses were tested. The first three simulations were based on hypotheses derived from random patterning processes, while the fourth tested pattern was based on a local density of cell fates. Patterns from all four simulations were significantly different from experimentally observed patterns^37^, in particular because the clustering of the cells sharing the same cell fate could not be reconciled with any of the tested models^37^.

The purpose of this study is to investigate to which amount the observed neighbourhood structure in ICM organoids can be explained by considering solely cell divisions. To this end, a 3D agent-based model is implemented. Agent-based models provide a technique to represent a wet-lab experiment under idealised conditions and are commonly used to study cancer growth, cell proliferation or the contribution of single cells towards collective cell migrations^41–47^. The model is given as a set of differential equations, describing mechanical cell–cell interaction, such as adhesion and repulsion forces, cell growth, and cell division involving cell fate heredity. It is assumed that the initial cell fate acquisition results in a random distribution at E3.5, which eventually leads to two segregated populations at E4.5^10,11,31,48^. Hence, the initial cell fate decision is modelled as a stochastic process (omitting a detailed description of the signalling pathway dynamics)^49–51^.

We use the model to investigate the hypothesis that the observed local cell fate clustering in 24 h old ICM organoids can arise from cell divisions alone, whereby cell fates are (partially) passed on to both daughter cells. Simulations were conducted under four hypotheses, each considering different cell fate switch rates during cell division. Our results indicate that the observed cell fate clustering can indeed be explained as a randomly distributed cell fate decision with subsequent divisions and cell fate heredity. Furthermore, based on the neighbourhood statistics, a time point for the cell fate decision (prior to the 24 h growth stage) can be inferred.

The core message of this article is that clustering of cell fates represents a transient state in ICM organoid development, which can arise solely from cell division and cell fate heredity. This transient stage occurs after an early cell fate decision and before cell sorting and does not exhibit significant temporal overlap with these processes.

## Results

**Figure 1:**
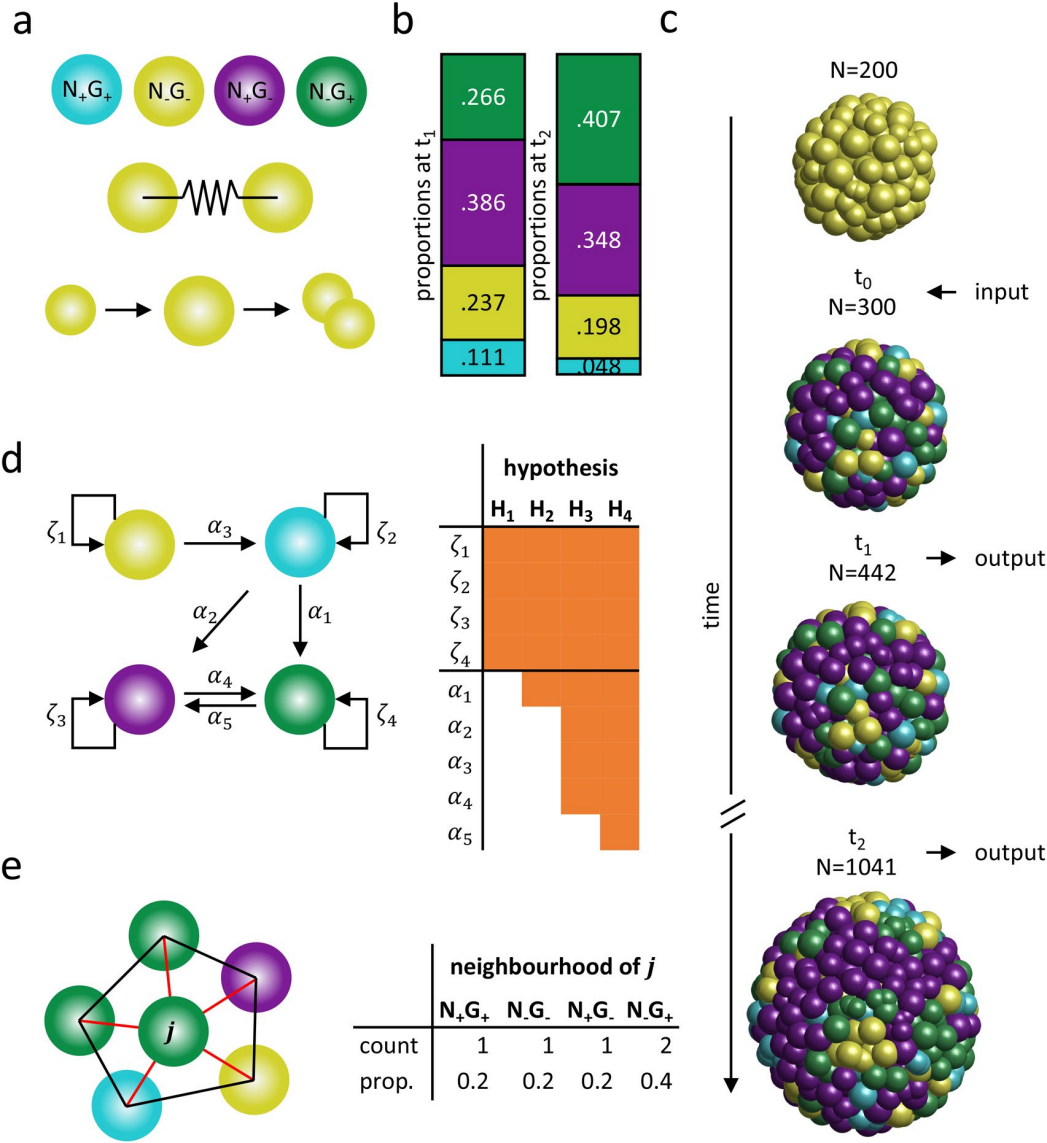
Outline of the mathematical model and the conducted analyses. (**a**) The model considers four different cell types (blue N_+_G_+_; yellow N_−_G_−_; purple N_+_G_−_; green N_−_G_+_). Neighbouring cells are connected via a force potential. Cells are growing over time and divide if they pass a given size. (**b**) The model utilises the proportions of cell types of 24 h old ICM organoids (left). The proportion of cell types of 48 h old ICM organoids (right). (**c**) The initial state of the model considers 200 undifferentiated cells. At a cell count of 200, 300 or 400 cells ($t_0$) the cells are randomly assigned to a cell type (based on the proportions of cell types in 24 h old ICM organoids. When the simulation reaches a cell count of 442 cells ($t_1$) and 1041 cells ($t_2$) the positions and types of the cells are saved. (**d**) Throughout the simulations, the cells pass on their cell types during cell division according to different hypotheses (H_1_–H_4_). The hypotheses increase in complexity i.e. the number of parameters. Parameter values are presented in Supplementary Table A1. (**e**) From the simulated results the neighbourhood statistics for each cell type are determined. Cell neighbours are identified via Delaunay triangulation. Red lines indicate neighbours of cell *j*. Black lines indicate neighbours not involving cell *j* (left). The neighbourhood structure of cell *j* is quantified and expressed as proportions of neighbouring cell types. This is performed for all cells and averaged over the four cell types (right).

**Figure 2:**
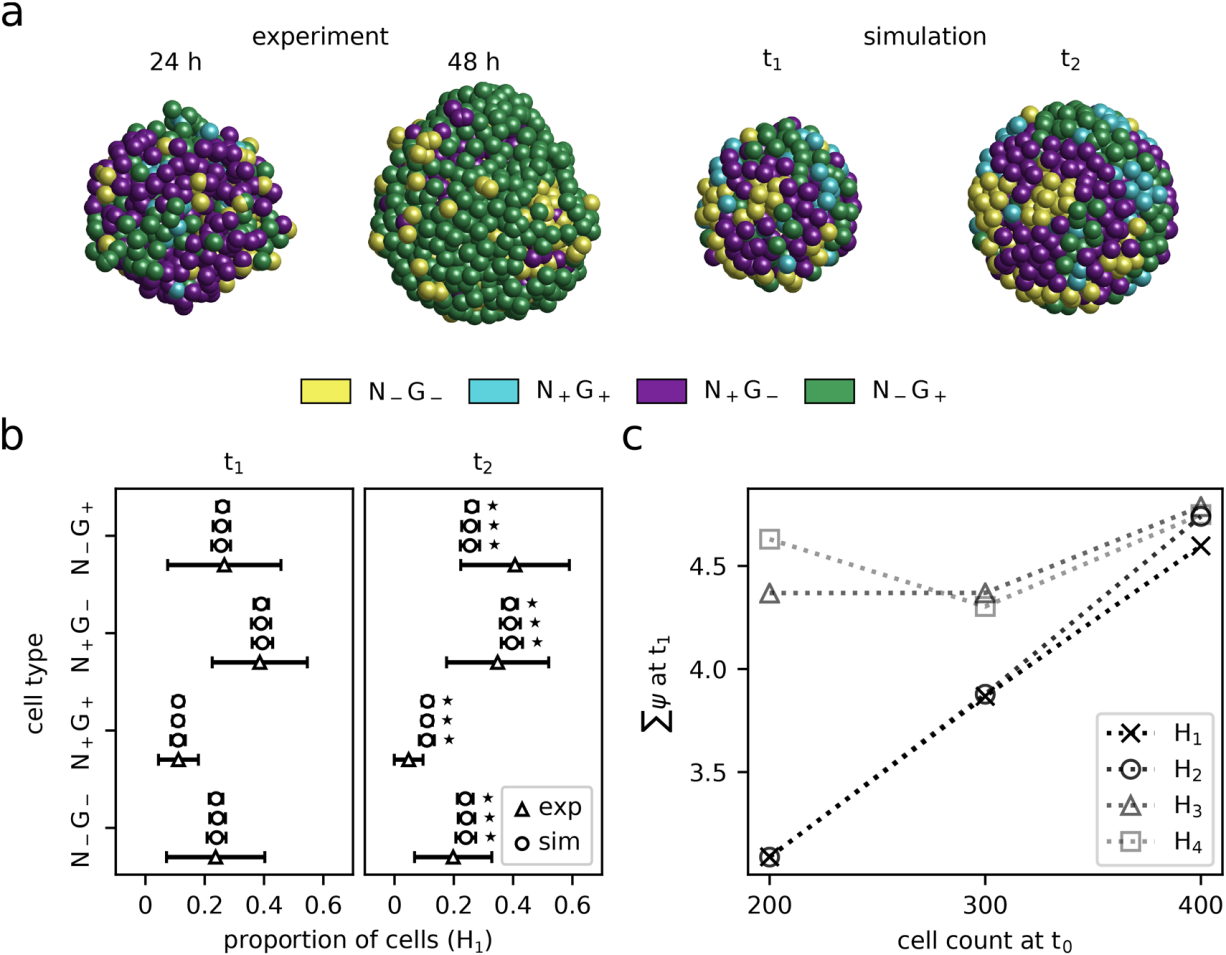
Expression type composition of ICM spheroids for H_1_. (**a**) ICM organoids (experimental data) for 24 h and 48 h and simulated ICM spheroids for $t_1$ and $t_2$. (**b**) Expression type composition of ICM organoids and ICM spheroids as percentage of the total number of cells within ICM organoids at $t_1$ and $t_2$. Simulations were performed under the assumption H_1_. Experimental data from Mathew et al. are indicated by triangles. Simulation results for different $t_0$ are indicated by circles. The error bars indicate the standard deviation. $t_0$ from lowest line to top: 200, 300 and 400 cells. Statistically significant differences between the cell fate proportion of ICM organoids and ICM spheroids are indicated by stars ($p < 0.05$; using a Wilcoxon–Mann–Whitney test with Bonferroni correction). (**c**) The effect size ($\psi$) as the relative deviation of the simulated and experimental neighbourhood statistics at $t_1$ for simulations performed under the assumptions H_1_, H_2_, H_3_ and H_4_.

**Figure 3:**
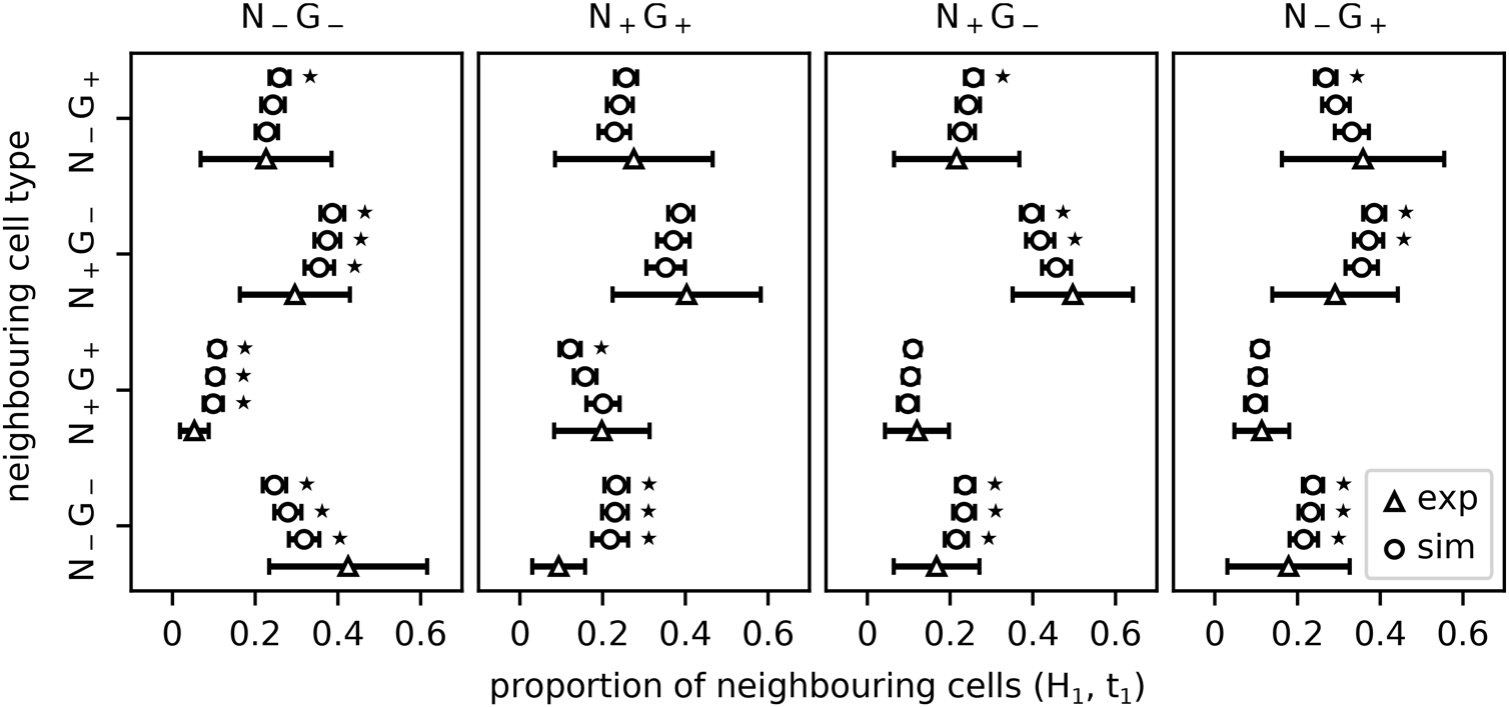
Expression type composition of neighbouring cells as percentage of the total of neighbouring cells at $t_1$. Simulations were performed under the assumption H_1_. Experimental data from Mathew et al. are indicated by triangles. Simulation results for different $t_0$ are indicated by circles. The error bars indicate the standard deviation. $t_0$ from lowest line to top: 200, 300 and 400 cells. Statistically significant differences between the neighbourhood structure of 24 h old ICM organoids and ICM spheroid patterns are indicated by stars ($p < 0.05$; using a Wilcoxon–Mann–Whitney test with Bonferroni correction).

We implemented an agent-based model in order to study to which extend the intricate 3D neighbourhood pattern in the ICM organoids are explained solely by cell proliferation. The model is based on qualitative and quantitative data derived from the experimental studies. In the experimental procedure^37^, the ICM organoids are assembled from 200 cells. This approach implies that the organoids of 200 cells exhibit a spatial positioning that can be assumed to be random. Initially, the cells in the ICM organoids coexpress both transcription markers (N_+_G_+_). Then, they downregulate one of the transcription factors to commit to the N_+_G_−_ or N_−_G_+_ cell fate. The proportions of the cell fates, as well as the 3D neighbourhood structures, are known for 24 h old ($t_1$) and 48 h old ($t_2$) ICM organoids.

The mathematical model considers four different cell fates, intercellular mechanics, as well as cell growth and proliferation, but omits intra- and intercellular chemical signalling as well as a cell sorting process (see Fig. 1a). The initial configuration of our model involves 200 undifferentiated cells. The initial cell type assignment is then set up as a process resulting in a random distribution of cell fates. Hereby, the cell type probabilities are fitted to the cell type proportions known from 24 h old ICM organoids (see Fig. 1b). An initial cell type assignment takes place before $t_1$. Testing different time points ($t_0$) for the cell fate assignment allows us to investigate the temporal resolution of this process. Thus, we assign the cell fates when the ICM spheroid reaches a cell count of 200, 300 or 400 cells (see Fig. 1c). During cell division, acquired cell fates are passed on to the daughter cells. We implemented four cell fate heredity strategies, formulated as hypothesis H_1_–H_4_ (see Fig. 1d) and tested their impact on the neighbourhood statistics. The model yields cell positions and the cell fates as the simulation reaches the cell counts at $t_1$ (approx. 442 cells) and $t_2$ (approx. 1041 cells, see Fig. 1c). Using these data, we quantify the neighbourhood statistics of the cells, given as the proportions of surrounding cell fates (see Fig. 1e). We compare this measure to the experimentally observed neighbourhood statistics.

One 24 h old ICM organoid, one 48 h old ICM organoid, as well as simulated ICM spheroids for $t_1$ and $t_2$, are shown in Fig. 2a. The model assumes for each cell fate the same constant division rate. The four hypotheses on the cell type heredity strategies increase in complexity. The transition probabilities for each hypothesis are fitted to reproduce the proportion of cell fates in 24 h old ICM organoids at $t_1$. In assumption H_1_, each cell passes on its cell fate to both daughter cells. However, in the experimental data, we observe a substantial increase in the proportions of N_−_G_+_ cells from $t_1$ to $t_2$, a phenomenon not captured by H_1_ (see Fig. 2b, Supplementary Table A2). Thus, we included asymmetric cell division in H_2_, such that N_+_G_+_ cells give rise to N_+_G_+_ and N_−_G_+_ cells. H_2_ reproduces the proportions of N_+_G_+_ cells at $t_2$, but does not cover the changes in the proportions of the other cell fates (see Supplementary Fig. A2a, Supplementary Table A2). Thus, we allowed additional cell fate transitions from N_−_G_−_ to N_+_G_+_, from N_+_G_+_ to N_+_G_−_ or N_−_G_+_, as well as from N_+_G_−_ to N_−_G_+_ in H_3_. H_4_ additionally allows a small flux between N_+_G_−_ and N_−_G_+_ cells. Both hypotheses reproduce the proportions of cell fates at $t_2$ better than H_1_ and H_2_ (see Supplementary Fig. A2b,c, Supplementary Table A2). In summary, with increasing complexity (i.e. allowing more cell fate switches, see Fig. 1d) the agreement between the simulated and experimental proportion data at $t_2$ increases.

We quantify the performance of the different hypotheses for the simulated neighbourhood statistics at $t_1$. To this end, we determine the effect size ($\psi$) as the relative deviation of the simulated from the experimental neighbourhood statistics at $t_1$ (see Eq. 7, Fig. 2c). H_1_ shows the best agreement with the data. H_2_ shows a similar agreement, while $\psi$ values for H_3_ and H_4_ indicate only poor agreement between simulated and experimental neighbourhood statistics. Given the determined $\psi$ values and the law of parsimony, we focus on the simulated results of H_1_.

Simulation results of the model hypotheses H_1_ agreed very well with the experimental data on the spheroid expression type composition, and could also explain the neighbourhood statistics to a large degree (see Fig. 3, Supplementary Tables A3, A4-A6 and Supplementary Fig. A3 for H_2_–H_4_). Overall the neighbourhood statistics agreed best with the experimental data if the cell fate assignment occurred at a cell count of 200 cells (i.e. directly after the formation of the ICM spheroids). Concerning the neighbourhood statistics measured at $t_2$, the prediction power of the model decreased strongly (see Supplementary Fig. A4, A5 and Supplementary Tables A3, A4–A6 for H_2_–H_4_). According to the overall comparison of the neighbourhood statistics, the best agreement between simulated and experimental data required $t_0=200$ cells (see Fig. 2c). A delayed cell fate assignment ($t_0 = 300$ or 400 cells) shifts the distribution of cell fates from a pronounced local clustering to a random distribution.

**Figure 4:**
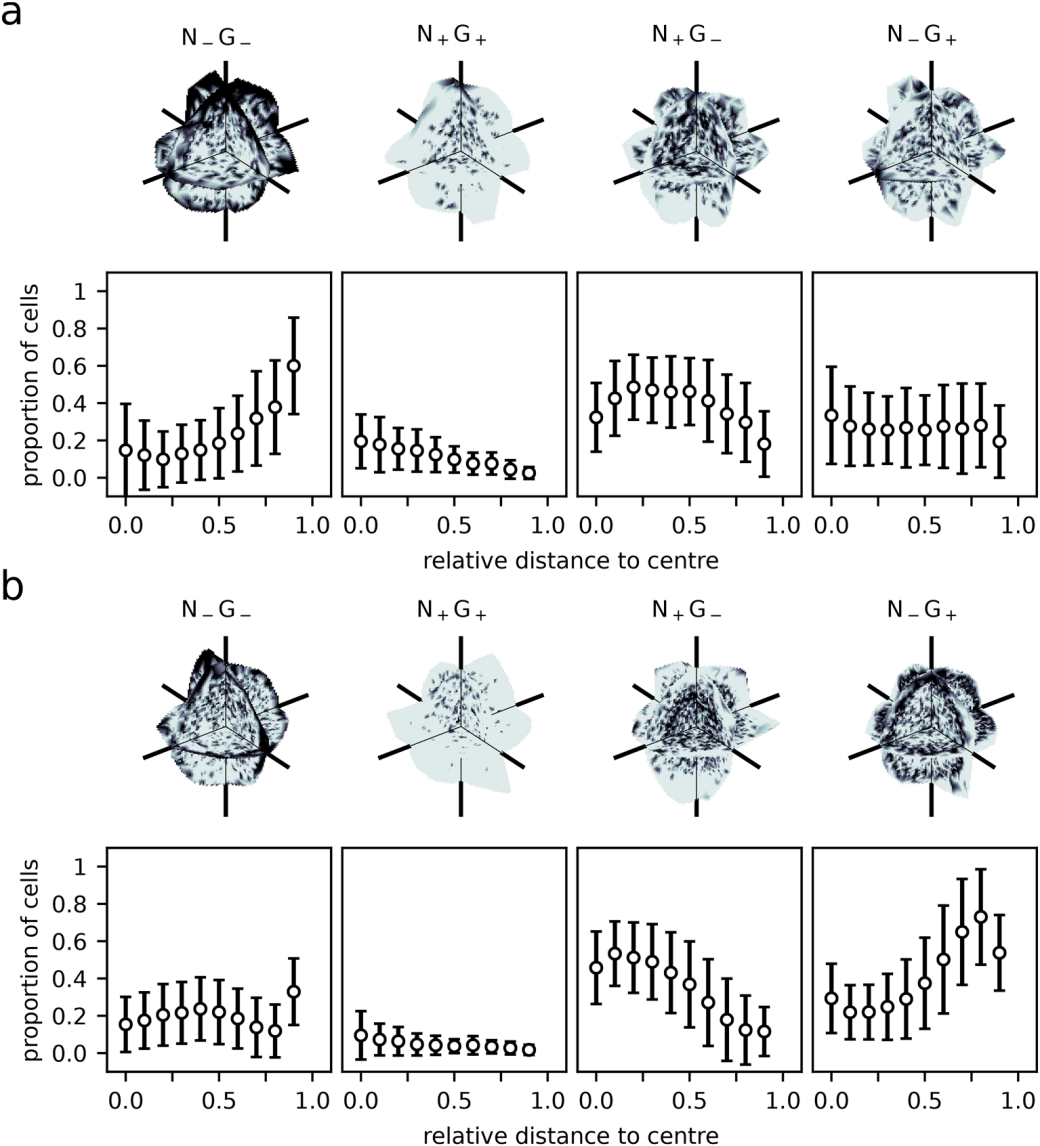
Expression type cluster analysis for ICM organoids. (**a**) 24 h old ICM organoids; (**b**) 48 h old ICM organoids. Black indicates the presence of an expression type, white indicates its absence, respectively. Shown are slices through the ICM organoids at cartesian origin. Expression type compositions in dependence of the relative distance to the ICM organoid centre. Cells are sorted according to their distance to the ICM organoid centre of mass and binned into 10 groups. Points indicate the average proportion of a cell fate type for the 10 bins, the bars denote the standard deviation.

The only disagreement between simulation and experiment concerned the neighbourhood statistics of N_−_G_−_ cells. In order to investigate the reasons for this disagreement, we conducted an additional spatial analysis of the expression type distribution. Figure 4 shows 3D views of the average ICM organoid composition for both time points $t_1$ and $t_2$, marking the spatial density of a given cell fate (see “Methods”). Cells with the expression type N_+_G_+_ were spread evenly over the whole ICM organoid at $t_1$ and $t_2$. The same distribution pattern was obtained for N_+_G_−_ and N_−_G_+_ expression type cells at $t_1$. At $t_2$, N_+_G_−_ cells formed a cluster in the centre of the ICM organoid while N_−_G_+_ cells formed an evenly distributed outer layer around the inner core of the ICM organoid. Concerning the N_−_G_−_ cells, we found that this expression type tended to be positioned in the outer parts of the ICM organoid at both time points, $t_1$ and $t_2$. In both cases, their distribution was unevenly spread over the outermost layer of cells in the ICM organoid.

## Discussion

Complex 3D spatial arrangements of cells with particular cell fates characterise early stages of PrE and Epi segregation. ICM organoids mimic those cell arrangements after 24 h of growth and after 48 h of growth for mid and late mouse blastocysts, respectively. Mathew et al.^37^ used a computational rule-based static model in order to reproduce the cell fate pattern measured in 24 h old ICM organoids. Hereby, four different rules for these cell fate assignments were tested, however, none of the rules could reproduce the observed neighbourhood pattern of local clustering in 24 h old ICM organoids^37^.

In this study, we investigated whether cell division and cell fate heredity alone can account for cell fate clustering and experimentally observed 3D neighbourhood statistics measured in 24 h old ICM organoids, resembling the ICM of the mid mouse blastocysts (E3.75). We demonstrated that a simple agent-based spheroid model, involving mechanical interactions such as adhesion and repulsion, cell division with cell fate heredity, and a stochastically driven cell fate, can explain the local clustering of cell fates in 24 h old ICM organoids.

The implemented model shows that an initial random cell fate distribution, in addition to cell division involving cell fate heredity, can explain the lineage composition and spatial distribution of N_+_G_−_ and N_−_G_+_ cells in 24 h old ICM organoids. The cell fate pattern in the ICM of the mid mouse blastocyst (E3.75) is investigated by several studies, highlighting the need of neighbourhood interactions for the rise of Epi and PrE lineages^26,28,46^. For instance, a comparable model, which is also based on displacement due to the physical forces and cell division, but takes also into account an intercellular network for cell fate decisions was used in Tosenberger et al. to predict cell fate decisions in in vivo mouse blastocysts^46^. According to their findings, the spatio-temporal distribution of PrE and Epi cells leads to a state in which Epi cells are preferentially surrounded by PrE cells and vice-versa^46^. However, we hypothesise that the local spatial clustering of cell fates as obtained in the 24 h old ICM organoids in Mathew et al. 2019 might not be indicative of specific neighbourhood interactions involved in cell fate decision^37^. Instead, we hypothesise that cell fate clustering is a transient state, characteristic of blastocysts and ICM organoids in the time span between cell fate decision and spatial sorting. In particular, our results indicate that the cell fate decision takes place prior to or only shortly after the organoids are assembled from 200 cells. The initial cell fate decision results in a random distribution. Subsequently, a phase of cell divisions can give rise to local cell clusters, exhibiting the same neighbourhood statistics as observed in 24 h old ICM organoids^37^.

With the given restrictions on the cell fate switches, it was not possible to fit the proportion transition from 24 to 48 h data (see Fig. 1b)^37^. Although cell fate switches are considered unlikely, we investigated if the proportion data and neighbourhood structures could be approximated better if further cell fate switches were allowed. Indeed, this relaxation allows for a better fit of the cell proportion data. However, the neighbourhood statistics are not approximated well under the hypotheses H_3_ and H_4_ (see Supplement). At $t_2$, N_−_G_+_ cells represented the majority in the ICM organoid. The increase in the relative amount of the N_−_G_+_ cells from $t_1$ to $t_2$ can be explained by either cell fate switches from N_+_G_−_ to N_−_G_+_ or by an enhanced cell division rate for N_−_G_+_ cells. The former can be considered as unlikely for two reasons: (1) the more cell fate changes are allowed within the model, the weaker was the agreement between simulated and experimentally observed neighbourhood patterns; (2) in the ICM, cell fate switches from N_+_G_−_ to N_−_G_+_ and vice versa are reported to be unlikely without an external stimulus^16,17,19^. A plausible mechanism for the latter can be an interaction between the cell growth rate and mechanical forces exerted by surrounding cells. Cells on the surface might grow faster than cells in the centre, where the cell density is high. At late stage blastocyst (E4.5), N_−_G_+_ expressing cells are predominantly found at the rim of ICM organoids where the cell density is lower, which might result in a higher cell division rate^25,53^. This mechanism appears plausible, and is well supported by studies reporting an interaction between the cell growth rate and mechanical forces^42,52^. A feedback of pressure onto cell growth is not included in our model, therefore, the model cannot reproduce the increase in the proportions of N_−_G_+_ cells from $t_1$ to $t_2$.

During blastocyst growth, PrE cells are sorted to the rim of the ICM, while Epi cells remain in the centre of the ICM^1,7,10–17^. This behaviour of PrE and Epi cells is reflected in ICM organoids. While N_+_G_−_ and N_−_G_+_ cells are evenly distributed over the 24 h old ICM organoid, they re-localise in 48 h old ICM organoids, forming a centre of N_+_G_−_ cells and a rim of N_−_G_+_ cells. N_+_G_+_ cells are co-expressing NANOG and GATA6, as described for early mouse blastocysts (E3.0)^7,11,12^. Thus, we expected them to be distributed evenly over the whole ICM for 24 h old and 48 h old ICM organoids, which was confirmed by the performed spatial analysis. For N_−_G_−_ cells, expressing low levels of NANOG and GATA6, we also expected a spatially homogeneous distribution. However, the spatial analysis revealed that N_−_G_−_ cells tended to be positioned at the rim of ICM organoids at the 24 h time point. This finding was surprising, but it explains the very high proportion of N_−_G_−_ cells in the neighbourhood of N_−_G_−_ cells. Our model assumptions treat all cell fates equally and do not involve a specific spatial positioning of one of the cell fates. Furthermore, we assume that the original cell fate distribution follows a random pattern (following from the experimental procedure to compose the initial organoids). Apparently, a subgroup of the N_−_G_−_ cells tends to assemble at the rim of the organoid. This behaviour has not been described in the literature before and is not yet understood. However, this positioning explains the disagreement between the expected neighbourhood statistics and the data for this cell type. The role and dynamics of N_−_G_−_ cells should be investigated in further studies.

One mechanism for efficient segregation of two cell types involves a difference in their physical properties^54,55^. For instance, Krupinski et al. hypothesised that the spatial segregation of Epi and PrE cells occurs partially due to differential adhesion^56^. Differential adhesion is a process also observed in in vitro embryonic stem cells, which relies on a different expression of adhesion molecules (e.g. E-cadherin) in the different cell types^57,58^. Even a modest difference in the expression level of a given cadherin is capable of leading to a cell sorting based on differential adhesion^59^. Filimonow et al. showed that no difference in E-cadherin levels between Epi and PrE cells can be found until E3.75^60^. However, Yanagida et al. showed that Epi and PrE cells are distinguishable by their expression of actin-cytoskeleton genes around E3.75–E4.5^61^, resulting in different mechanical properties of the cells and an increased motility of PrE cells compared to Epi cells^61^. Increased cell motility supports cell sorting by allowing the cells to sample for their preferred neighbourhood. Using a mathematical model, they showed that a differential cell-cell affinity can lead to a cell segregation within one round of cell division^61^. These studies support our findings very well. While cell division and cell fate heredity can explain the cell type clustering at $t_1$ very well, the mismatch between the simulated and experimental data at the 48 h time point indicates the onset of a cell sorting phase between $t_1$ and $t_2$. Since we did not implement a cell sorting process, the model cannot reproduce the segregated neighbourhood statistics at t2.

ICM organoids represent an in vitro model of the in vivo ICM of the mouse blastocyst. They mimic key events and timings of the ICM in mouse blastocysts. Thus, we expect the findings of our model to be translatable to the in vivo process of ICM formation. Taken together, our results, in agreement with the results of Filimonow et al. and Yanagida et al. indicate that the cells mechanical properties differentiate around E3.75^60,61^, allowing a cell sorting mechanism to act between E3.75 to E4.0. Our model points out that the observed cell fate clusters in 24 h old ICM organoids (reflecting E3.75 in the mouse embryo) do not require a cell sorting mechanism. Instead, cell fate clustering arises in response to cell division and associated stable cell fate inheritance from pre-specified progenitors. We, therefore, argue in favour of a temporal separation of the four phases: (1) coexpression of the cell fate markers NANOG and GATA6; (2) cell fate commitment; (3) local cell fate clustering and (4) cell sorting.

## Methods

### Experimental data

Our study is based on experimental results from Mathew et al.^37^. They used a system based on mouse embryonic stem cells which allows differentiation into primitive endoderm like cells^27^. After induction of differentiation, 200 of these cells were placed in a non-adhesive well and centrifuged. Subsequently, the cells formed the 3D ICM organoids. This experimental approach implies that the cell fates in the initial organoid, composed of about 200 cells, are randomly distributed. In 24 h and 48 h old ICM organoids, expression levels for NANOG and GATA6, as well as the nuclei positions for all cells were determined by fluorescence microscopy and subsequent image analysis. In total, four different expression types were established: NANOG and GATA6 expressing cells (double positive; N_+_G_+_), cells that express both transcription factors at a low level (double negative; N_−_G_−_), cells that express NANOG at a high level and GATA6 at a low level (N_+_G_−_) and cells that express GATA6 at a high level and NANOG at a low level (N_−_G_+_). In the following, the stage of 24 h old and 48 h old ICM organoids will be referred to as $t_1$ and $t_2$, respectively. During the simulations, cell fates and positions were recorded when the cell count in the simulation coincided with the average ICM organoid cell count in the experiment at $t_1$ (441 cells at 24 h) and $t_2$ (1041 cells at 48 h).

### Model implementation

An individual cellbased model that defines a small set of cellular features, implemented by Stichel et al.^47^, is extended to investigate the rise of local cell fate clustering in ICM organoids. The model describes the displacement of cells in response to external forces exerted by surrounding cells:

1 $$\begin{aligned} \mathbf {F}_{i,k} = F_0 \cdot F(r_i, r_k, ||\mathbf {x}_i-\mathbf {x}_k||)\cdot \frac{\mathbf {x}_i-\mathbf {x}_k}{||\mathbf {x}_i-\mathbf {x}_k||}, \end{aligned}$$

with $F_0=1$ a positive constant, representing the strength of the mechanical interaction, $r_i$ the radius of the *i*th cell, $\mathbf {x}_i = (x_i, y_i, z_i)$ the position of the *i*th cell and

2 $$\begin{aligned} F(r_i, r_k, d) = {\left\{ \begin{array}{ll} 2\cdot (e_{-2a(d-(r_i+r_k))}-e_{-a(d-(r_i+r_k))}) &{} d < \sigma r_i \\ 0 &{} d \ge \sigma r_i\end{array}\right. }. \end{aligned}$$

As given by the Morse potential (Eq. 2), the force between two cells is positive (repulsive), if the distance between the cell centres is below the sum of their radii, and negative (attractive) for $(r_i + r_k)< d < \sigma r_i$ ($\sigma =4$). Repulsion accounts for the limited compressibility of cells, while attraction accounts for cell–cell adhesion. The attractive part of the potential is cut off for cells at a distance above $\sigma r_i$. If the distance of two cells equals the sum of their radii they are in perfect distance and thus neither exert attraction nor repulsion onto each other. The parameter $a=0.6$ describes the spatial decay of the interaction force, and together with the parameter $F_0$ determines the elasticity of the cells. Displacement of cells in this model is only determined by forces exerted on them:

3 $$\begin{aligned} \frac{d\mathbf {x}_i}{dt} = \sum _{k,k\ne i} \mathbf {F}_{i, k}. \end{aligned}$$

Each cell in this model is described by three features. A position $\mathbf {x}$, a radius *r* and an expression type $\epsilon$. Other model parameters are assumed to be the same for all cells (e.g. elasticity, adhesion strength). The radius (size) of the cells is growing over time with

4 $$\begin{aligned} \frac{dr_i}{dt} = k\cdot (r_*-r_i), \end{aligned}$$

with $k=1$ a (positive) growth constant and the maximum cell size $r_*=1$. Cell division is determined by a stochastic process which depends on the cell radius but not on the cell type. During cell division, the cell volume is preserved. The mother cell keeps its position ($\mathbf {x}_m$) and reduces its radius ($r_m$) with

5 $$\begin{aligned} r_{m, new} = r_m \cdot \root 3 \of {\frac{1}{2}}. \end{aligned}$$

The daughter cell ($\mathbf {x}_d$) is generated close to $\mathbf {x}_m$, with $\mathbf {x}_d = \mathbf {x}_m + \mathbf {\xi }$ with $\mathbf {\xi }$ a random 3D vector ($\delta _x, \delta _y, \delta _z$) containing small values ($\delta<< r_{m,new}$). The daughter cell is assigned the same size as the mother cell ($r_d = r_{m, new}$). The factor conserves the total cell volume during cell division. Directly after cell division, both cells are growing as given by Eq. (4) and change their positions as given by Eq. (3).

Since it was shown that the initial cell fate decision can be described as a stochastic process^49–51^ and because the experimental approach leads to a random assembly of cell types, the initial expression type $\epsilon$ of the simulated cells is assigned randomly from the four expression types

6 $$\begin{aligned} \epsilon \in \{\text {N}_{-}\text {G}_{-}, \text {N}_{+}\text G_{+}, \text {N}_{+}\text G_{-}, \text {N}_{-}\text G_{+}\}. \end{aligned}$$

### Cell fate heredity

The model is used to test several assumptions addressing cell fate heredity. In total, four different hypotheses are tested. The different hypotheses increase in complexity and are designed to reproduce the experimentally measured, spatially not resolved proportions of cell fates in ICM organoids at $t_1$ and $t_2$. In hypothesis H_1_, each cell passes on its cell fate to both daughter cells. For hypothesis H_2_, N_+_G_+_ are allowed to give rise to N_+_G_+_ or N_−_G_+_ cells. In H_3_ the rules of H_2_ are extended such, that cell fate switches from N_+_G_−_ to N_−_G_+_ are allowed. H_4_ additionally considers a small flux between N_+_G_−_ and N_−_G_+_ cells.

### Model framework

The implemented model is illustrated by a flowchart in Supplementary Fig. A1. For all hypotheses, the initial cell fate proportion has been determined and assigned to occur at an earlier time point ($t_0$). In particular, we couple $t_0$ to the cell count and initiate the cell fate when the simulated ICM spheroid reached a cell count of 200, 300 or 400 cells. We determine the initial proportions of the cell fates by proportion data at $t_1$ taken from the experimental data, omitting the spatial component. Using the system of linear differential equations (A1–A3), the theoretical proportions at $t_0$ are determined. After cell fate acquisition, the simulation proceeds until $t_1$ and $t_2$ are reached. Both time points are coupled to cell counts as well (i.e. 441 cells at $t_1$ and 1041 cells at $t_2$). When $t_1$ and $t_2$ are reached, the model saves the spatially resolved cell positions and cell fates as output. Each simulation is repeated 100 times.

### Neighbourhood analysis and statistics

Cell neighbours were determined for both, simulation and experimental data, using the Delaunay triangulation. For the neighbourhood statistics, we derived the set of all neighbours of all cells of a given fate *j*, and computed the proportion of cell types based on this neighbourhood set. Neighbourhood proportions were collected for every executed simulation. For statistical comparison between experimental and simulated data according to single neighbourhood structures, we used the Wilcoxon–Mann–Whitney test with Bonferroni correction for multiple testing. In order to compare the overall fit of the simulated pattern to experimental data, we used the effect size as the relative deviation ($\psi$) as given in Ref.^37^

7 $$\begin{aligned} \psi =\frac{|(\bar{s}-\bar{m})|}{\bar{m}}, \end{aligned}$$

with $\bar{s}$ representing the mean of the simulated data and $\bar{m}$ the mean of the experimental data.

### Spatial analysis of expression type distribution

During visual inspection of the biological data, we noticed that N_−_G_−_ cells often clustered at the rim of the ICM organoids. In order to visualise this effect, the following procedure was applied to experimental data for 24 h old and 48 h old ICM organoids. We assumed the spatial heterogeneity to be the same in small and in large ICM organoids, and therefore we normalised the size of all ICM organoids to equalise them in space. For normalisation, the median absolute divergence is used instead of the standard deviation because it is more robust with respect to outliers. The centre of mass for double negative cells was determined and the entire ICM organoid was rotated such that the centre of mass was located on the x-axis (i.e. $x>0, y=0, z=0$). The rotated cell positions were combined in one data set. Each double negative cell was assigned the value 1 and the value 0 was assigned to all other cells. Interpolation on the combined data generated a continuous clustering pattern from experimental data. In the interpolated dataset, the label 1 indicated the presence of a particular expression type, while 0 indicated its absence. The procedure was repeated for N_+_G_+_, N_+_G_−_ and N_−_G_+_ cells to show that the visualised heterogeneity is not an artefact resulting of this procedure. In order to analyse the cell fate proportions in dependence on their relative distance to the ICM organoid centre, we measured the distance of the cells of each ICM organoid to their respective centre of mass. Subsequently, we divided these distances into ten intervals. Eventually the mean proportions and standard deviations for the ten intervals were determined for all 24 h old ICM organoids and 48 h old ICM organoids. Unless otherwise stated, the model and data analysis methods were implemented in MATLAB R2019b. The model is available via GitHub (https://github.com/TimLiebisch/ICM_spheroids).

## Supplementary information

Supplementary Information.
