# Supplementary material for: Cell fate clusters in ICM organoids arise from cell fate heredity and division: a modelling approach
Source: Sci Rep. 2020 Dec 29;10:22405. doi: 10.1038/s41598-020-80141-3 (PMC7772343; doi:10.1038/s41598-020-80141-3)
Supplement: Supplementary file 1 — Supplementary Information. [file 41598_2020_80141_MOESM1_ESM.pdf]

Cell Fate Clusters in ICM Organoids Arise from Cell Fate Heredity & Division – A Modelling Approach Supplemental Material

Tim Liebisch[1,*], Armin Drusko[1], Biena Mathew[2], Ernst HK Stelzer[2], Sabine C Fischer[3], and Franziska Matthäus[1]

[1]Faculty of Biological Sciences & Frankfurt Institute for Advanced Studies (FIAS), Goethe Universität Frankfurt am Main, Ruth-Moufang-Straße 1, Frankfurt 60438, Germany
[2]Faculty of Biological Sciences & Buchmann Institute for Molecular Life Sciences (BMLS), Goethe Universität Frankfurt am Main, Max-von-Laue Str. 15, Frankfurt 60438, Germany
[3]Center for Computational and Theoretical Biology (CCTB), Julius-Maximilians-Universität Würzburg, Campus Hubland Nord 32, 97074 Würzburg, Germany
[*]liebisch@fias.uni-frankfurt.de

ABSTRACT

During the mammalian preimplantation phase, cells undergo two subsequent cell fate decisions. During the first decision, the trophectoderm and the inner cell mass are formed. Subsequently, the inner cell mass segregates into the epiblast and the primitive endoderm. Inner cell mass organoids represent an experimental model system, mimicking the second cell fate decision. It has been shown that cells of the same fate tend to cluster stronger than expected for random cell fate decisions. Three major processes are hypothesised to contribute to the cell fate arrangements: 1) chemical signalling; 2) cell sorting; and 3) cell proliferation. In order to quantify the influence of cell proliferation on the observed cell lineage type clustering, we developed a mechanical agent-based model accounting for mechanical cell-cell interaction, i.e. adhesion and repulsion, cell division, stochastic cell fate decision and cell fate heredity. The model supports the hypothesis that initial cell fate acquisition is a stochastically driven process, taking place in the early development of inner cell mass organoids. Further, we show that the observed neighbourhood structures can emerge solely due to cell fate heredity during cell division.

Flowchart

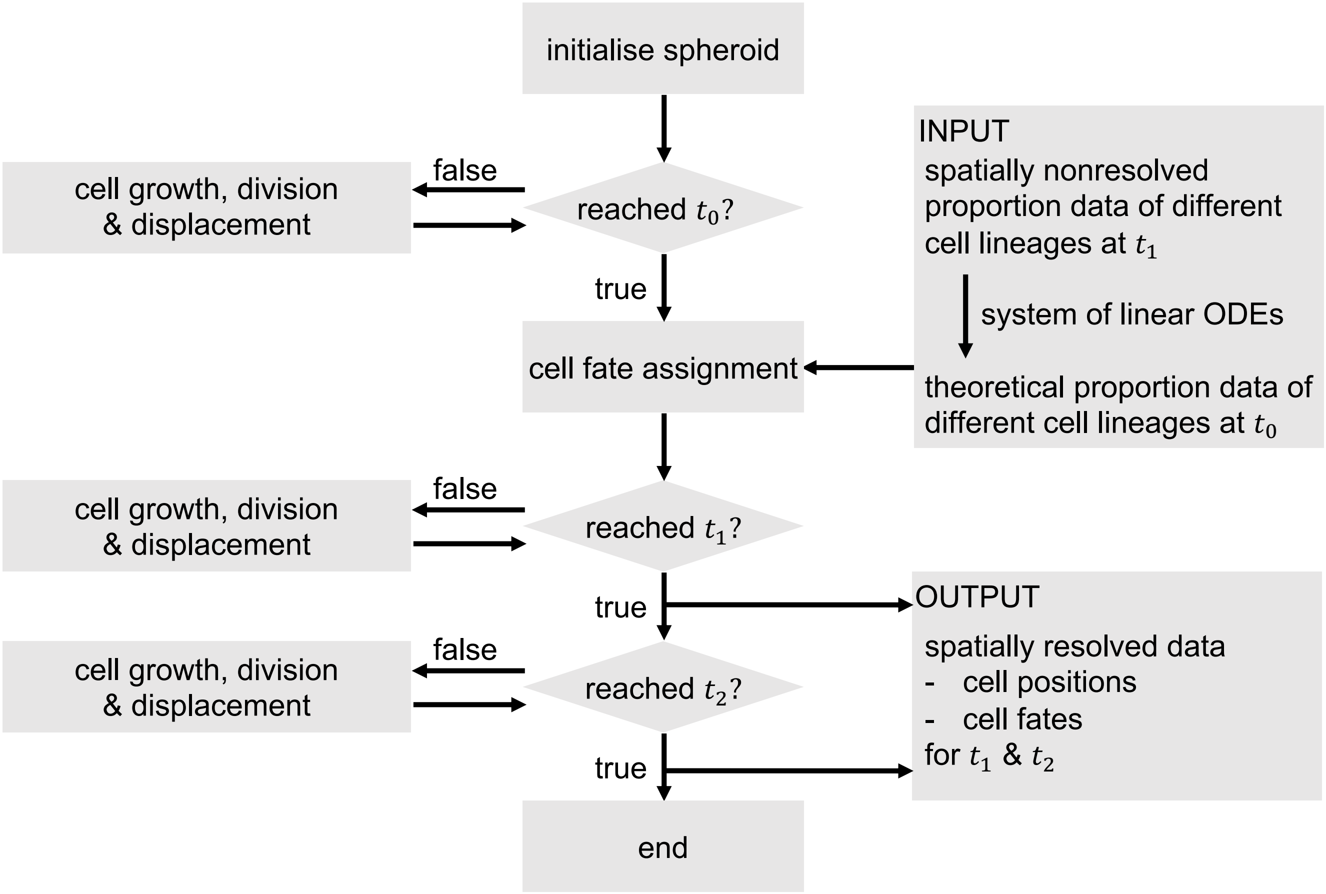

Figure A1. Flowchart of the implemented model.

Parameter estimation for different hypotheses

The model can be used to test several assumptions addressing cell fate heredity. In total, four different hypotheses are tested. Each hypothesis is considering cell fate switches during cell division according to Fig. 1d. During cell division, the cell fate is passed on to the daughter cells. A cell fate switch is possible with a given rate. The model considers the following cell fate switches: N_-G_- remain N_-G_- (ζ_1) or become N_+G_+ (α_3). N_+G_+ remain N_+G_+ (ζ_2) or become N_+G_- (α_1) or N_-G_+ (α_2). N_+G_- and N_-G_+ remain N_+G_- (ζ_3) and N_-G_+ (ζ_4) or switch to the opposite cell fate (α_4) and (α_5), respectively. These cell fate transitions form a system of linear ordinary differential equations, which can be written as

$$d\mathbf{f}/dt = \mathbf{Af} \tag{A1}$$

$$\begin{pmatrix} dN_+G_+/dt \\ dN_-G_-/dt \\ dN_+G_-/dt \\ dN_-G_+/dt \end{pmatrix} = \begin{pmatrix} \zeta_2 - \alpha_1 - \alpha_2 & \alpha_3 & 0 & 0 \\ 0 & \zeta_1 - \alpha_3 & 0 & 0 \\ \alpha_1 & 0 & \zeta_3 - \alpha_4 & \alpha_5 \\ \alpha_2 & 0 & \alpha_4 & \zeta_4 - \alpha_5 \end{pmatrix} \cdot \begin{pmatrix} N_+G_+ \\ N_-G_- \\ N_+G_- \\ N_-G_+ \end{pmatrix}, \tag{A2}$$

with the analytical solution:

$$\mathbf{f}(t) = c_{N_+G_+}\mathbf{v}_{N_+G_+}e^{\lambda_{N_+G_+}t} + c_{N_-G_-}\mathbf{v}_{N_-G_-}e^{\lambda_{N_-G_-}t} + c_{N_+G_-}\mathbf{v}_{N_+G_-}e^{\lambda_{N_+G_-}t} + c_{N_-G_+}\mathbf{v}_{N_-G_+}e^{\lambda_{N_-G_+}t}, \tag{A3}$$

with $\mathbf{v}$ and λ the eigenvectors and eigenvalues of the coefficient-matrix $\mathbf{A}$, respectively. The unknown prefactor c values can be determined by inserting the known cell counts of the different cell types at t_1. The rates for the different cell fate switches vary for the different hypotheses (see Tab. A1).

Table A1. Cell fate transition rates for the four different hypotheses.

	**hypothesis**			
	**H_1**	**H_2**	**H_3**	**H_4**
ζ_1	1	1	1	1
ζ_2	1	1	1	1
ζ_3	1	1	1	1
ζ_4	1	1	1	1
α_1	0	1	0.7863	0.7863
α_2	0	0	0.7863	0.7863
α_3	0	0	0.2123	0.2123
α_4	0	0	0.2997	0.3927
α_5	0	0	0	0.1

Hypothesis 2, 3 and 4

The neighbourhood statistics for H_2 agreed reasonably well with the experimental data (see Fig. A3). The neighbourhood pattern of cells with the expression types N_-G_- and N_+G_- at t_1 were similar to the neighbourhood structures obtained under assumption H_1, including the misfit involving N_-G_- cells. In addition, simulated N_+G_+ cells were significantly less often neighbours of other N_+G_+ cells than the experimental data suggests (see Tab. A4). The latter speaks against a cell fate change of N_+G_+ cells into N_-G_+ cells. If performed under the assumption of the H_2 model, the evaluated proporitons for t_2 showed a better agreement with the experimental data. In particular, the predicted neighbourhood statistics of N_+G_+ cells differ statistically less substantially from the *in vitro* measured proportions compared to the simulated cell neighbourhood statistics under the assumption H_1 (see. Fig. A2 and Tab. A2).

If we consider cell fate switches from N_+G_- to N_-G_+ and vice versa, which according to the literature are considered as unlikely, a third and a fourth hypothesis can be formulated. In both hypotheses, the strong increase in the amount of N_-G_+ is explained by cell fate switches from N_+G_- to N_-G_+. While H_3 permits only the cell fate switches from N_+G_- to N_-G_+, H_4 is considering a small flux between both cell fates. The parameter values for both hypotheses are shown in Tab. A1. As expected, the simulated proportions for H_3 and H_4 agree very well with the experimental data at t_1 and t_2 (see. Fig. A2 and Tab. A2).

Comparing H_3 and H_4 to H_1 and H_2, we observe that their ψ values are higher, thus the quality of the fit of the neighbourhood statistics is lower (see. Fig. 2c). The neighbourhood distributions largely agree with experimental data at t_1 for the neighbourhood of double positive and NANOG positive cells. The simulated proportions of GATA6 positive cells adjacent to other GATA6 positive cells are significantly lower compared to experimental data, independent of the cell count of the ICM spheroid at which the initial cell fate is determined (see. Fig. A3 and Tab A5 and A6).

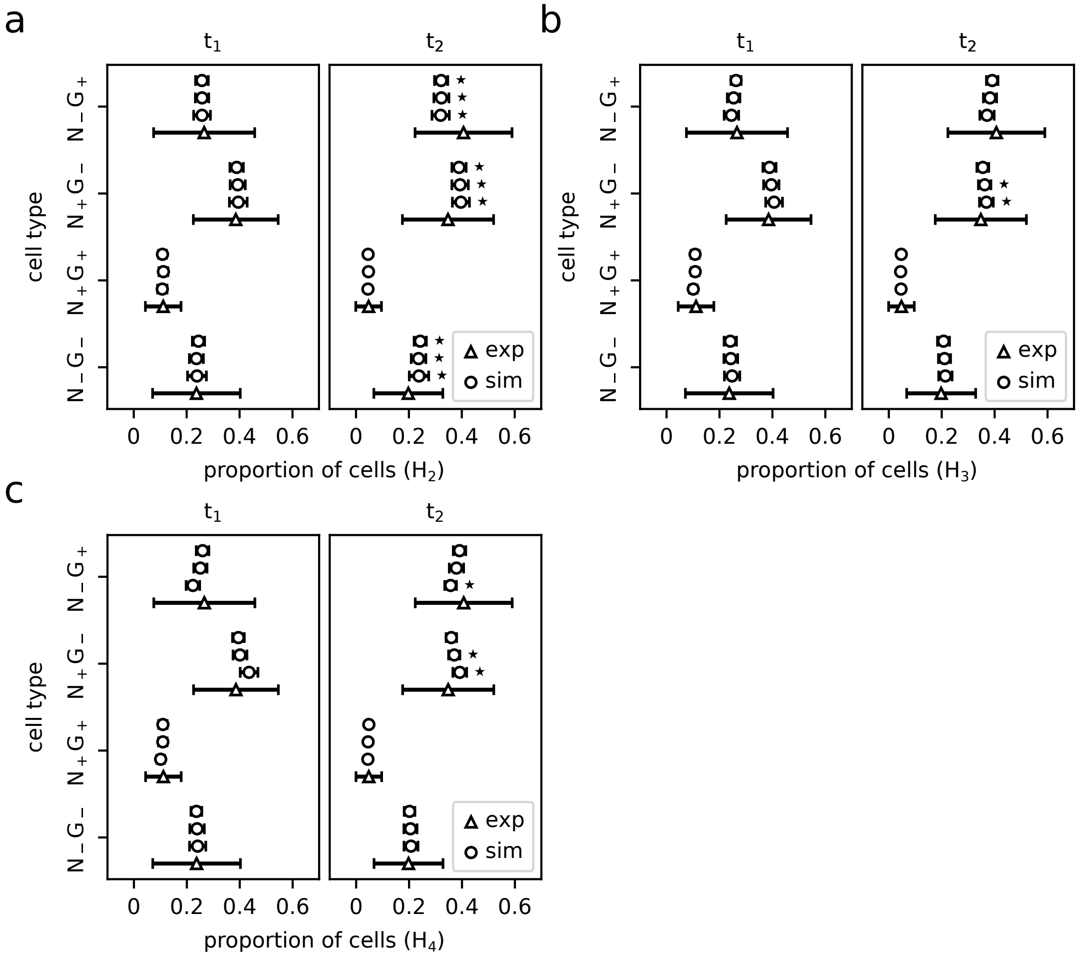

Figure A2. Expression type composition of ICM spheroids for H_2, H_3 and H_4. Expression type composition of ICM organoids and ICM spheroids as percentage of the total number of cells within ICM organoids at t_1 and t_2. Simulations were performed under the assumption H_2 (a), the assumption H_3 (b) and the assumption H_4 (c). Experimental data from Mathew *et al.* (2019) are indicated by triangles. Simulation results for different t_0 are indicated by circles. The error bars indicate the standard deviation. t_0 from lowest line to top: 200, 300 and 400 cells. Statistically significant differences between the cell fate proportion of ICM organoids and ICM spheroids are indicated by stars ($p < 0.05$; using a Wilcoxon-Mann-Whitney test with Bonferroni correction).

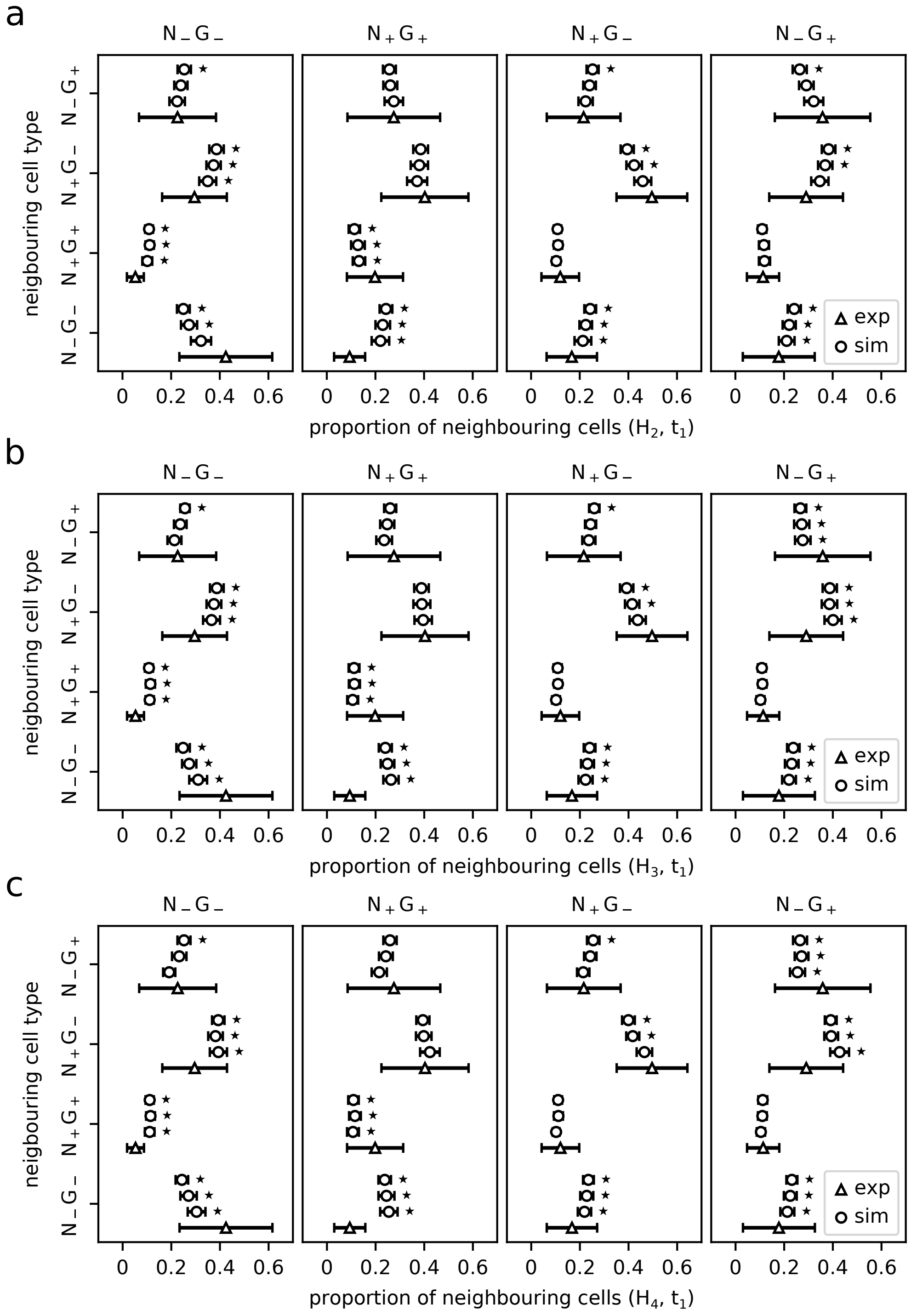

Figure A3. Expression type composition of neighbouring cells as percentage of the total of neighbouring cells at t_1. Simulations were performed under the assumption H_2 (a), the assumption H_3 (b) and the assumption H_4 (c). Experimental data from Mathew *et al.* (2019) are indicated by triangles. Simulation results for different t_0 are indicated by circles. The error bars indicate the standard deviation. t_0 from lowest line to top: 200, 300 and 400 cells. Statistically significant differences between the neighbourhood structure of 24 h old ICM organoids and ICM spheroid patterns are indicated by stars ($p < 0.05$; using a Wilcoxon-Mann-Whitney test with Bonferroni correction).

Expression type composition of neighbouring cells at t_2

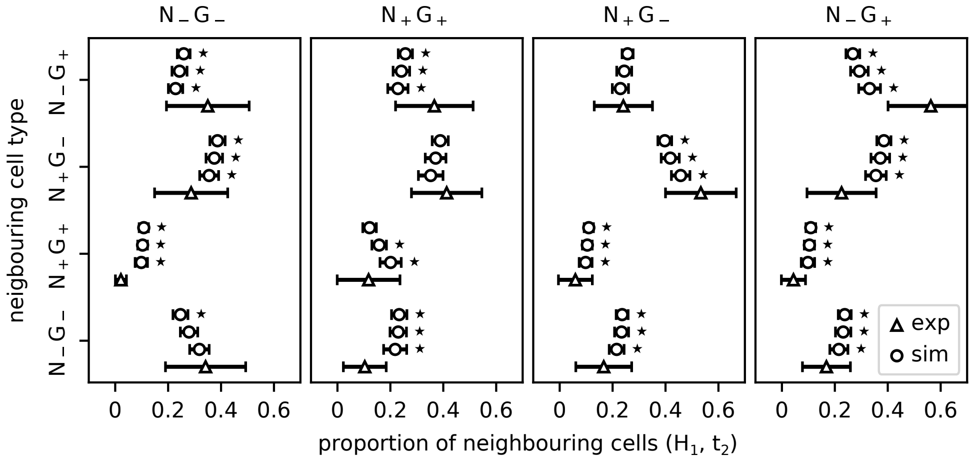

Figure A4. Expression type composition of neighbouring cells as percentage of the total of neighbouring cells at t_2. Simulations were performed under the assumption H_1. Experimental data from Mathew *et al.* (2019) are indicated by triangles. Simulation results for different t_0 are indicated by circles. The error bars indicate the standard deviation. t_0 from lowest line to top: 200, 300 and 400 cells. Statistically significant differences between the neighbourhood structure of 24 h old ICM organoids and ICM spheroid patterns are indicated by stars ($p < 0.05$; using a Wilcoxon-Mann-Whitney test with Bonferroni correction).

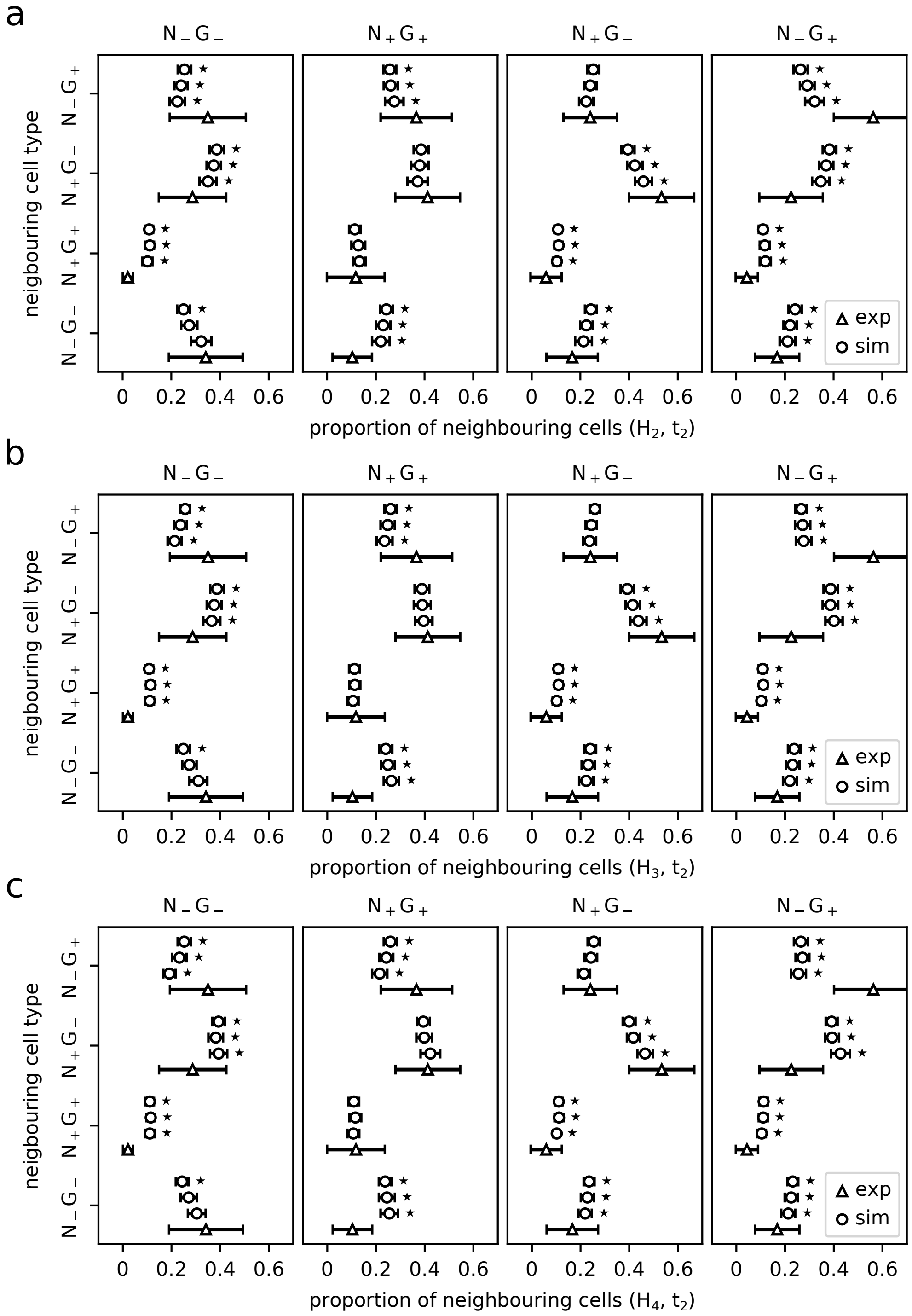

Figure A5. Expression type composition of neighbouring cells as percentage of the total of neighbouring cells at t_2. Simulations were performed under the assumption H_2 (a), the assumption H_3 (b) and the assumption H_4 (c). Experimental data from Mathew *et al.* (2019) are indicated by triangles. Simulation results for different t_0 are indicated by circles. The error bars indicate the standard deviation. t_0 from lowest line to top: 200, 300 and 400 cells. Statistically significant differences between the neighbourhood structure of 24 h old ICM organoids and ICM spheroid patterns are indicated by stars ($p < 0.05$; using a Wilcoxon-Mann-Whitney test with Bonferroni correction).

p-values

Table A2. p-values for the statistical comparison of the simulated expression type composition of ICM spheroids to the experimental data at t_1 and t_2. Simulations were performed under the assumption H_1, the assumption H_2, the assumption H_3 and the assumption H_4. For statistical comparison a Wilcoxon-Mann-Whitney test with Bonferroni correction is performed. Statistically significant differences between simulated and experimental data are indicated in red ($p < 0.05$).

		t_1				t_2			
neighbouring expression type	cell count at t_0	H_1	H_2	H_3	H_4	H_1	H_2	H_3	H_4
N_-G_+	400	0.240	0.345	0.150	0.244	8e-8	3e-4	0.207	0.190
	300	0.399	0.325	0.638	0.624	8e-8	3e-4	0.087	0.070
	200	0.468	0.340	0.955	0.208	9e-8	2e-4	0.031	0.006
N_+G_-	400	0.675	0.645	0.685	0.775	4e-4	5e-4	0.027	0.018
	300	0.743	0.822	0.888	0.992	6e-4	3e-4	0.012	0.005
	200	0.935	0.941	0.727	0.053	4e-4	3e-4	0.006	3e-4
N_+G_+	400	0.978	0.767	0.713	0.896	4e-12	0.039	0.023	0.021
	300	0.910	0.955	0.771	0.908	5e-12	0.023	0.048	0.045
	200	0.927	0.747	0.311	0.355	1e-11	0.051	0.028	0.059
N_-G_-	400	0.240	0.061	0.068	0.083	0.002	0.001	0.042	0.067
	300	0.399	0.078	0.061	0.070	0.001	0.002	0.032	0.049
	200	0.468	0.072	0.526	0.067	0.001	0.002	0.021	0.043

Table A3. p-values for the statistical comparison of the simulated expression type composition of neighbouring cells of ICM spheroids to the experimental data at t_1 and t_2. Simulations were performed under the assumption H_1. For statistical comparison a Wilcoxon-Mann-Whitney test with Bonferroni correction is performed. Statistically significant differences between simulated and experimental data are indicated in red ($p < 0.05$). Not statistically significant differences between experimental data and simulated expression type compositions of neighbouring cells for H_1 (excluding N_-G_- cells) with $t_0 = 200$ cells are indicated in blue.

neighbouring expression type	cell count at t_0	t_1 expression type				t_2 expression type			
		N_-G_-	N_+G_+	N_+G_-	N_-G_+	N_-G_-	N_+G_+	N_+G_-	N_-G_+
N_-G_+	400	6.9e-4	0.388	0.001	0.001	9.4e-5	1.8e-6	0.230	4.4e-16
	300	0.005	0.154	0.018	0.018	7.5e-6	3.0e-7	0.993	4.7e-14
	200	0.030	0.091	0.144	0.144	3.8e-7	4.7e-8	0.312	1.1e-11
N_+G_-	400	1.1e-8	0.963	6.7e-5	2.5e-5	3.3e-11	0.895	5.2e-14	9.9e-12
	300	1.7e-8	0.506	9.1e-4	6.0e-4	5.0e-10	0.137	1.4e-10	1.1e-11
	200	1.2e-5	0.189	0.059	0.007	1.1e-7	0.004	3.0e-4	2.7e-11
N_+G_+	400	5.0e-13	1.5e-4	0.480	0.560	8.0e-21	0.036	6.2e-10	6.4e-16
	300	3.1e-11	0.061	0.240	0.248	1.3e-20	3.8e-5	3.8e-9	5.6e-15
	200	1.8e-9	0.625	0.092	0.116	4.4e-20	1.1e-8	7.6e-8	2.4e-13
N_-G_-	400	1.3e-7	4.4e-15	01.8e-4	8.3e-7	0.001	8.8e-15	1.6e-5	1.6e-6
	300	1.7e-5	5.6e-15	0.008	1.7e-6	0.081	1.3e-14	2.4e-5	8.7e-6
	200	0.002	2.7e-14	0.144	3.0e-5	0.936	1.0e-13	4.3e-4	3.2e-4

Table A4. p-values for the statistical comparison of the simulated expression type composition of neighbouring cells of ICM spheroids to the experimental data at t_1 and t_2. Simulations were performed under the assumption H_2. For statistical comparison a Wilcoxon-Mann-Whitney test with Bonferroni correction is performed. Statistically significant differences between simulated and experimental data are indicated in red ($p < 0.05$).

neighbouring expression type	cell count at t_0	t_1 expression type				t_2 expression type			
		N_-G_-	N_+G_+	N_+G_-	N_-G_+	N_-G_-	N_+G_+	N_+G_-	N_-G_+
N_-G_+	400	0.001	0.405	0.003	01.5e-4	4.7e-5	1.9e-6	0.435	2.4e-16
	300	0.006	0.496	0.023	0.005	5.0e-6	3.6e-6	0.862	3.6e-14
	200	0.061	0.943	0.297	0.139	2.7e-7	3.8e-5	0.141	3.5e-12
N_+G_-	400	9.9e-9	0.919	6.5e-5	3.4e-5	3.4e-11	0.775	3.6e-14	3.8e-12
	300	1.2e-7	0.751	0.001	6.7e-4	3.0e-10	0.429	1.3e-9	1.6e-11
	200	4.0e-5	0.539	0.074	0.018	4.4e-7	0.146	5.9e-4	5.0e-11
N_+G_+	400	1.1e-13	1.5e-5	0.352	0.627	8.3e-21	0.099	1.1e-9	3.8e-16
	300	6.6e-14	3.5e-4	0.523	0.870	7.6e-21	0.015	4.8e-10	9.6e-17
	200	6.8e-11	0.002	0.175	0.656	1.9e-20	0.008	6.1e-9	5.4e-17
N_-G_-	400	2.1e-7	1.4e-15	7.9e-10	3.6e-7	0.002	3.1e-15	3.7e-6	3.2e-7
	300	8.1e-6	4.0e-15	3.0e-8	1.1e-5	0.052	9.5e-15	8.1e-5	9.5e-5
	200	0.005	1.6e-14	1.4e-6	5.1e-5	0.982	4.8e-14	5.8e-4	7.6e-4

Table A5. p-values for the statistical comparison of the simulated expression type composition of neighbouring cells of ICM spheroids to the experimental data at t_1 and t_2. Simulations were performed under the assumption H_3. For statistical comparison a Wilcoxon-Mann-Whitney test with Bonferroni correction is performed. Statistically significant differences between simulated and experimental data are indicated in red ($p < 0.05$).

neighbouring expression type	cell count at t_0	t_1 expression type				t_2 expression type			
		N-G-	N+G+	N+G-	N-G+	N-G-	N+G+	N+G-	N-G+
N-G+	400	7.9e-4	0.472	6.4e-4	1.3e-4	7.5e-5	2.5e-6	0.159	2.9e-16
	300	0.008	0.228	0.012	4.4e-4	2.2e-6	7.5e-7	0.993	1.4e-15
	200	0.191	0.108	0.048	8.3e-4	3.4e-8	1.3e-7	0.543	2.7e-15
N+G-	400	8.8e-9	0.996	3.5e-5	1.9e-5	6.2e-11	0.939	2.0e-14	7.0e-12
	300	9.7e-8	0.927	3.9e-4	2.4e-5	3.4e-10	0.932	2.6e-11	5.8e-12
	200	1.2e-6	0.771	0.008	1.4e-6	7.3e-9	0.823	2.4e-7	2.9e-12
N+G+	400	3.1e-13	1.1e-5	0.402	0.490	6.2e-21	0.119	8.4e-10	8.0e-16
	300	2.1e-14	2.2e-5	0.413	0.649	6.7e-21	0.092	6.5e-10	4.0e-16
	200	5.6e-14	2.3e-6	0.148	0.205	7.0e-21	0.181	8.3e-9	6.9e-15
N-G-	400	2.4e-7	1.9e-15	1.3e-9	9.7e-7	0.001	3.6e-15	5.6e-6	1.4e-6
	300	7.9e-6	1.1e-15	7.6e-9	2.2e-6	0.043	2.0e-15	3.5e-5	8.5e-6
	200	0.001	4.5e-16	7.5e-8	1.3e-5	0.676	8.3e-16	1.1e-4	1.1e-4

Table A6. p-values for the statistical comparison of the simulated expression type composition of neighbouring cells of ICM spheroids to the experimental data at t_1 and t_2. Simulations were performed under the assumption H_4. For statistical comparison a Wilcoxon-Mann-Whitney test with Bonferroni correction is performed. Statistically significant differences between simulated and experimental data are indicated in red ($p < 0.05$).

neighbouring expression type	cell count at t_0	t_1 expression type				t_2 expression type			
		N-G-	N+G+	N+G-	N-G+	N-G-	N+G+	N+G-	N-G+
N-G+	400	0.002	0.455	0.002	1.9e-4	4.3e-5	2.5e-6	0.292	5.1e-16
	300	0.021	0.164	0.018	3.3e-4	1.0e-6	2.9e-7	0.993	1.2e-15
	200	0.886	0.036	0.724	6.0e-5	1.5e-9	6.4e-9	0.024	6.1e-17
N+G-	400	3.5e-9	0.846	9.2e-5	5.0e-6	6.8e-12	0.701	1.5e-13	7.5e-12
	300	2.6e-8	0.620	5.8e-4	4.7e-6	8.8e-11	0.636	3.7e-11	5.0e-12
	200	8.3e-9	0.134	0.128	1.7e-8	3.6e-11	0.101	0.002	1.8e-13
N+G+	400	7.1e-14	6.2e-6	0.484	0.728	7.6e-21	0.155	6.0e-10	2.8e-16
	300	1.5e-14	2.6e-5	0.645	0.645	7.0e-21	0.073	3.1e-10	7.4e-16
	200	1.5e-13	2.6e-6	0.126	0.265	6.5e-21	0.163	7.5e-9	2.6e-15
N-G-	400	5.6e-8	2.4e-15	2.8e-9	1.4e-6	6.3e-4	5.2e-15	1.8e-6	8.5e-6
	300	7.9e-6	1.8e-15	1.7e-8	5.1e-6	0.031	3.0e-15	4.7e-5	4.5e-5
	200	5.8e-4	1.2e-15	1.8e-7	4.4e-5	0.517	1.6e-15	2.7e-4	5.1e-4
